# Structure–Function Modulation of Antarctic Krill Protein via Maillard Glycosylation with Mono- and Polysaccharides

**DOI:** 10.3390/foods15091497

**Published:** 2026-04-25

**Authors:** Linjing Huang, Weixin Ke, Chunbao Li, Danchen Aaron Yang

**Affiliations:** 1 College of Veterinary Medicine, Nanjing Agricultural University, Nanjing 210095, China; huanglinjing@stu.njau.edu.cn (L.H.);; 2 College of Food Science and Technology, Nanjing Agricultural University, Nanjing 210095, China; chunbao.li@njau.edu.cn

**Keywords:** Antarctic krill protein, Maillard glycation, monosaccharides, polysaccharides, particle size, emulsifying properties, water- and oil-holding capacity

## Abstract

Antarctic krill protein (AKP) was conjugated with three reducing monosaccharides (ribose, glucose, fructose) and five polysaccharides (xanthan gum, konjac glucomannan, inulin, κ-carrageenan, and pectin) via a controlled Maillard-type glycation process (pH 7.0, 90 °C, 24 h). We comparatively evaluated glycation reactivity (color change and degree of glycation), structural responses (particle size, FTIR, intrinsic fluorescence, surface hydrophobicity, and microstructure), and key techno-functional properties (solubility, water- and oil-holding capacities, and emulsifying performance). Monosaccharide-conjugated AKP exhibited stronger browning and higher apparent glycation activity, consistent with the higher reactivity of small-molecule sugars. In contrast, polysaccharide-conjugated AKP showed more pronounced improvements in dispersion-related and interfacial functions, reflecting enhanced steric stabilization and hydration after polysaccharide grafting. Notably, κ-carrageenan conjugation delivered the strongest overall functional enhancement (water-holding capacity ≈ 22.1 g/g; oil-holding capacity ≈ 10 g/g) and the most stable emulsions. These findings clarify how glycosylating-agent size and architecture steer AKP glycation outcomes, providing a practical basis for tailoring AKP ingredients for aqueous and emulsion-based foods.

## 1. Introduction

Antarctic krill (*Euphausia superba*) is among the most abundant marine bioresources [1,2]. In current industrial practice, krill is primarily valorised for lipid fractions (e.g., krill oil), while large amounts of protein-rich biomass remain underutilised [3]. Antarctic krill protein exhibits high nutritional quality and potential functional application value. Specifically, it contains all essential amino acids (EAAs) required by the human body, with EAAs accounting for more than 40% of the total amino acid content, meeting the FAO/WHO recommendations for high-quality dietary proteins [4,5]. It is particularly rich in lysine and leucine, which are essential for muscle protein synthesis and tissue repair, while glutamic acid and aspartic acid are present in relatively high amounts, contributing to metabolic regulation and nitrogen balance [6,7]. These compositional features further highlight the nutritional value and functional potential of AKP as a high-quality protein source. However, native AKP typically exhibits poor solubility and pronounced aggregation, which undermines its processing performance in aqueous dispersions and limits its effectiveness in gel and emulsion systems [8,9]. Consequently, developing a food-grade, scalable approach to improve AKP dispersibility and interfacial functionality is essential for broadening its applications beyond conventional lipid extraction streams.

Protein glycation via the Maillard reaction offers a practical route to tune protein structure and functionality without introducing synthetic reagents. In this non-enzymatic process, carbonyl groups at the reducing ends of sugars react with free amino groups on proteins to form covalent protein–carbohydrate conjugates, accompanied by a cascade of reaction products [10,11]. Appropriately controlled glycation can increase hydrophilicity and steric repulsion, reshape surface charge and hydrophobicity, and thereby influence solubility, interfacial adsorption, and water/oil binding [12]. Importantly, the molecular size, conformation, and architecture of the glycosylating agent are expected to govern reaction kinetics as well as the resulting conjugate structures, thereby shaping the routes to functional improvement.

The influence of the glycosylating agent on Maillard-type modification has been demonstrated in a variety of protein systems. For example, glycation of cod skin collagen peptides with xylose significantly improved emulsifying and foaming properties [13], whereas modification of bovine serum albumin with glucose or mannose enhanced foaming capacity but markedly reduced emulsifying performance [14]. Carrageenan modification of fish gelatin altered its molecular conformation and led to the formation of a more viscous gel [15]. Similarly, glycation of goat whey protein with gum arabic or citrus pectin improved solubility, emulsifying performance, and foaming properties [16]. Collectively, these studies suggest that carbohydrate type plays a decisive role in determining the balance between glycation reactivity and functional reinforcement. In general, monosaccharides react more readily because of their small molecular size and easier access to protein amino groups, often resulting in stronger browning and higher apparent grafting. Polysaccharides, by contrast, usually show lower reactivity but may generate bulkier, more highly hydrated conjugates that contribute to steric stabilization, viscosity enhancement, or network-mediated functional improvement [17,18]. However, for marine proteins, and especially for AKP, available evidence remains limited and is often confined to one or two carbohydrates, making it difficult to disentangle the respective contributions of covalent grafting, hydration effects, and macromolecular architecture.

To address this gap, we selected three monosaccharides with different carbon skeletons and five polysaccharides with clearly distinct structural and physicochemical characteristics. The five polysaccharides—xanthan gum, konjac glucomannan, inulin, carrageenan, and pectin—were chosen not merely because they are common food-grade hydrocolloids, but because they represent structurally diverse polysaccharide systems. Xanthan gum is a high-molecular-weight, relatively rigid polysaccharide with high viscosity [19]; konjac glucomannan is a flexible, highly hydrophilic neutral polysaccharide with strong water-binding capacity [20]; inulin is a lower-molecular-weight fructan with a comparatively shorter chain structure; carrageenan is a sulfated polysaccharide carrying negative charges [21]; and pectin is an anionic polysaccharide with a branched architecture and good hydration properties. Because Maillard-type glycation depends not only on the presence of reactive groups but also on the spatial accessibility and interaction between reactants, these differences were expected to affect glycation behavior in different ways. Specifically, polysaccharides with larger molecular size or greater chain rigidity may impose stronger steric hindrance and reduce the accessibility of protein amino groups, whereas smaller or more flexible chains may facilitate molecular contact and conjugation [22,23]. In addition, charged polysaccharides such as carrageenan and pectin may further modulate electrostatic interactions, aggregation behavior, and interfacial rearrangement of the resulting conjugates [24]. We therefore hypothesized that these structurally diverse carbohydrates would differentially regulate AKP conformation, aggregation, and interfacial behavior, ultimately leading to distinct techno-functional outcomes.

By systematically comparing these eight glycosylating agents, this study aimed to establish structure–function links that explain (i) how sugar type controls glycation reactivity and AKP conformational changes, and (ii) how these changes translate into dispersion-related properties and emulsification performance. The resulting evidence provides practical guidance for selecting glycosylating agents to tailor AKP for different application targets, ranging from improved dispersibility to enhanced emulsion stability.

## 2. Materials and Methods

### 2.1. Materials

Frozen shelled Antarctic krill meat used in this study was purchased from Liaoyu Group Co., Ltd. (Dalian, China). Three monosaccharides, including ribose (150.13 Da), glucose (180.16 Da), and fructose (180.16 Da) and five polysaccharides, including xanthan gum (Mw ≈ 2 × 10^6^–2 × 10^7^ Da), konjac glucomannan (Mw ≈ 6 × 10^5^–2 × 10^6^ Da), inulin (Mw ≈ 2–5 × 10^3^ Da), κ-carrageenan (Mw ≈ 1 × 10^5^–1 × 10^6^ Da), and pectin (Mw ≈ 5 × 10^4^–3 × 10^5^ Da), were purchased from Yuanye Biological Technology Co., Ltd. (Shanghai, China). All other reagents were of analytical grade. A bicinchoninic acid (BCA) assay kit used for protein quantification was purchased from Solarbio Science & Technology Co., Ltd. (Beijing, China).

### 2.2. Preparation of Antarctic Krill Protein (AKP)

The extraction of Antarctic krill protein refers to the method of Li et al. [25] with slight modifications. The thawed krill meat was homogenized with deionized water at a solid-to-liquid ratio of 1:5 (*w*/*v*) using a high-speed homogenizer (PD500, PRIMA Technology Co., Ltd., London, UK) in an ice bath to prevent heat-induced protein degradation. The slurry pH was adjusted to 12.0 with NaOH (2 mol/L) and stirred at 4 °C for 30 min to promote protein solubilization. After centrifugation at 8000× *g* for 10 min at 4 °C (Avanti J-26S XP, Beckman Coulter, Brea, CA, USA), the supernatant was collected and the pH was adjusted to 4.5 using 2 mol/L HCl to reach the isoelectric point of krill proteins, facilitating protein precipitation. The mixture was then allowed to stand at 4 °C for 30 min to complete precipitation, followed by centrifugation at 8000× *g* for 10 min. The precipitate was redissolved, adjusted to pH 7.0, dialysed (4 °C, 24 h), freeze-dried (48 h), and stored at −80 °C until use. The protein content of the extracted AKP was determined by the Kjeldahl method.

### 2.3. Glycation of AKP with Mono- and Polysaccharides

Glycosylation modification of AKP according to the method of Zeng et al. [26]. AKP was mixed with each sugar (ribose, glucose, fructose, xanthan gum, konjac glucomannan, inulin, carrageenan, and pectin) at a mass ratio of 1:1 (protein: sugar, *w*/*w*) in distilled water and adjusted to pH 7.0. Dispersions were hydrated overnight at 4 °C to ensure complete hydration, then heated at 90 °C for 24 h to promote Maillard-type glycation and the reaction was terminated by rapid cooling in an ice bath. The mixtures were dialyzed at 4 °C for 24 h to remove unreacted small molecules, followed by freeze-drying and storage at −80 °C. Antarctic krill protein samples were labeled based on the type of added sugar: ribose (A-RIB), fructose (A-FRU), glucose (A-GLC), konjac glucomannan (A-KGM), xanthan gum (A-XAN), carrageenan (A-CAR), inulin (A-INU), and pectin (A-PEC).

### 2.4. Color Measurement

Color measurements of protein powders were performed using a CR-400 portable colorimeter (Konica Minolta, Tokyo, Japan). Prior to analysis, the instrument was calibrated using a standard white calibration plate according to the manufacturer’s instructions to ensure measurement accuracy. Protein powders were gently filled into a clean, dry sample holder and carefully compacted using a flat spatula to obtain a smooth and uniform powder, minimizing the influence of surface roughness and light scattering. The sample surface was leveled to ensure consistent optical conditions. The measurement aperture was placed in full contact with the sample surface, and measurements were performed under standardized illumination conditions. For each sample, five independent readings were taken at randomly selected but evenly distributed positions to minimize local heterogeneity. Color parameters are recorded as L* (lightness), a* (redness), and b* (yellowness). The total color difference (ΔE*) was calculated using the following equation [27]:

(1) $$\Delta E^{*}\;=\;\sqrt{{\left(\Delta L^{*}\right)}^{2}+{\left(\Delta a^{*}\right)}^{2}+{\left(\Delta b^{*}\right)}^{2}}$$

### 2.5. Degree of Glycation (DG)

According to the method of Rao et al. [28], the o-phthalaldehyde (OPA) method was used to analyze free amino groups for quantifying protein samples. Protein solutions (2 mg/mL) were freshly prepared in distilled water and gently mixed to ensure complete dispersion, followed by hydration at 4 °C overnight. The OPA reagent was freshly prepared prior to analysis by dissolving 80 mg o-phthalaldehyde in 1 mL methanol, followed by sequential addition of 5 mL of 20% (*w*/*v*) sodium dodecyl sulfate (SDS), 50 mL of 0.1 mol/L borate buffer, and 200 μL β-mercaptoethanol, and then diluting to 100 mL with ultrapure water. The reagent was protected from light and used immediately after preparation. For the assay, 200 μL of protein solution was mixed with 4 mL of freshly prepared OPA reagent and incubated at 35 °C for 2 min under dark conditions. Absorbance was measured at 340 nm using a microplate reader (Infinite M200 PRO, Tecan Trading AG, Männedorf, Switzerland). The degree of grafting (DG) was calculated as:

(2) $$\mathrm{DG}\;(\%)\;=\;\frac{A_{0}-A_{t}}{A_{0}}\;\times \;100$$

where A_0_ and A_t_ are the absorbance values before and after glycosylation, respectively.

### 2.6. Structural Characterization

#### 2.6.1. Particle Size Distribution

Hydrated protein dispersions (10 mg/mL, *w*/*v*) were prepared by gently stirring in deionized water to ensure uniform dispersion, followed by overnight hydration at 4 °C. Prior to measurement, the dispersions were gently vortexed to redisperse any settled particles. Particle size distribution was determined according to the method of Xie et al. [29], using a Mastersizer 3000 laser diffraction particle size analyzer (Malvern Panalytical, Malvern, UK). Before measurement, clean the system using the instrument’s cleaning function, then perform a background measurement. Deionized water was used as the dispersant, the refractive index of the dispersant was set to 1.33, and the refractive index and absorption of the sample were set to 1.52 and 0.001, respectively. The obscuration level was maintained between 5% and 15% to ensure optimal signal quality. Add the sample manually according to the instrument’s instructions until the obscuration reaches the required range, then start the measurement. Each sample is measured five times. All measurements were performed at room temperature, and particle size was expressed as the volume-weighted mean diameter (D_[4,3]_).

#### 2.6.2. FTIR Analysis of Secondary Structure

Protein secondary structure was evaluated using a Fourier Transform Infrared (FTIR) spectrometer (Thermo, Rockford, IL, USA). Measurements were performed according to the method of Liu et al. [30]; protein samples were mixed with potassium bromide (KBr) at a mass ratio of 1:100, ground thoroughly in an agate mortar, and pressed into pellets for analysis. Before analyzing the samples, the spectrum of KBr was measured as a background signal. Spectra were collected from 4000 to 400 cm^−1^ at a resolution of 4 cm^−1^ with 64 scans accumulated. Data were collected and further analyzed in the 1700–1600 cm^−1^ range (Amide I region) using OMNIC 9.2 and PeakFit 4.12 software.

#### 2.6.3. Intrinsic Fluorescence

Intrinsic fluorescence spectra were measured according to the method of Li et al. [31] with slight modifications. Protein solutions were prepared at a concentration of 1 mg/mL in distilled water and allowed to equilibrate at 4 °C overnight to ensure complete hydration. Prior to measurement, the samples were equilibrated to room temperature (~25 °C) and gently mixed to ensure homogeneity. Aliquots of the protein solutions were transferred into a quartz microplate for analysis. Fluorescence spectra were recorded using a SpectraMax M2e microplate reader (Molecular Devices, Sunnyvale, CA, USA) with an excitation wavelength of 280 nm and an emission scan range of 325–450 nm. Both excitation and emission slit widths were set to 4 nm.

#### 2.6.4. Surface Hydrophobicity (H_0_)

Protein surface hydrophobicity (H_0_) was determined using 1-anilinonaphthalene-8-sulfonic acid (ANS) as a fluorescent probe, following Zheng et al. [32]. Protein solutions (0.8 mg/mL) were prepared and diluted to 0.2, 0.4, and 0.8 mg/mL. ANS solution (20 μL, 8 mmol/L) was added to 1 mL of each diluted protein solution. After vortexing, the mixtures were incubated for 15 min at room temperature in the dark. Fluorescence intensity was measured at 375 nm (excitation) and 485 nm (emission) (Molecular Devices, Sunnyvale, CA, USA). Protein concentration was plotted against fluorescence intensity, and the slope of the linear regression was taken as H_0_.

#### 2.6.5. Microstructure by SEM

The microstructural characteristics of freeze-dried samples were observed using scanning electron microscopy (SEM) (ZEISS Sigma 300, Carl Zeiss AG, Jena, Germany). The pre-prepared freeze-dried protein samples were mounted on aluminum stubs using conductive adhesive and labeled accordingly, followed by sputter-coating with a thin layer of gold to enhance conductivity. Imaging was performed using a secondary electron detector (SE2) under an accelerating voltage of 5 kV, with a working distance of 9.5–9.9 mm. Micrographs were acquired at a magnification of 100×.

### 2.7. Functional Characterization

#### 2.7.1. Solubility

Protein solubility was determined according to the method of Grossmann et al. [33], with slight modifications. Briefly, 10 mg of protein powder was accurately weighed and dispersed in distilled water to obtain a final concentration of 5 mg/mL. The dispersion was gently stirred to ensure uniform suspension and then allowed to hydrate at 4 °C overnight. After hydration, the samples were centrifuged at 8000× *g* for 15 min at 4 °C using a refrigerated centrifuge (Avanti J-26S XP, Beckman Coulter, CA, USA). The supernatant was carefully collected without disturbing the precipitate. The protein concentration in the supernatant was determined using a bicinchoninic acid (BCA) assay kit with bovine serum albumin (BSA) as the standard. A calibration curve was constructed using BSA solutions of known concentrations, following the manufacturer’s instructions. Protein solubility (%) was calculated as the ratio of soluble protein (in the supernatant) to the total protein content in the initial sample.

#### 2.7.2. Water- and Oil-Holding Capacity

Water-holding capacity (WHC) and oil-holding capacity (OHC) were determined using a gravimetric method as described previously [34], with slight modifications. Briefly, 0.5 g of protein powder was accurately weighed into pre-weighed centrifuge tubes, followed by the addition of 10 mL of deionized water or sunflower oil. The mixtures were vortexed thoroughly to ensure complete dispersion and then allowed to equilibrate at 25 °C for 1 h to facilitate sufficient water/oil absorption. After equilibration, the samples were centrifuged at 3000× *g* for 5 min at room temperature. The supernatant was carefully decanted, and the tubes were inverted for 1 min to remove residual unbound liquid. The tubes containing the hydrated or oil-bound protein were then reweighed. WHC and OHC were calculated as the mass of water or oil retained per unit mass of protein (g/g).

#### 2.7.3. Emulsifying Activity and Stability

The emulsifying properties of protein samples were evaluated using the emulsifying activity index (EAI) and emulsion stability index (ESI) according to a previously reported method [35], with slight modifications. Protein solutions (5 mg/mL) were prepared in deionized water and allowed to fully hydrate at 4 °C overnight prior to analysis. For emulsion preparation, 20 mL of protein solution was mixed with 5 mL of sunflower oil (4:1, *v*/*v*, aqueous phase: oil phase). The mixture was homogenized using a high-speed homogenizer (PD500, PRIMA Technology Co., Ltd., London, UK) at 10,000 rpm for 1 min at room temperature (~25 °C) to form a coarse emulsion. Immediately after homogenization (0 min) and after standing for 10 min, 50 μL aliquots were withdrawn from the bottom of the emulsion and diluted into 5 mL of 0.1% (*w*/*v*) sodium dodecyl sulfate (SDS) solution to prevent further droplet aggregation. The absorbance of the diluted emulsion was measured at 500 nm using a UV–visible spectrophotometer. The calculation of EAI and ESI is based on the following formulas:

(3) $$\mathrm{EAI}\;(m^{2}/g)\;=\;\frac{2\times 2.303\times A_{0}\times N}{C\times \varphi \times 10,000}$$

(4) $$\mathrm{ESI}\;(\%)=\frac{A_{10}\times 100}{A_{0}}$$

where N is the dilution factor, A_0_ and A_10_ are the absorbance values at 0 and 10 min, respectively, C is the protein concentration (g/mL), and φ is the oil-to-water phase ratio.

### 2.8. Statistical Analysis

Each experiment was repeated five times (n = 5), and the results are presented as mean ± standard deviation. Differences among groups were evaluated using one-way analysis of variance (ANOVA). Assumptions were assessed on the basis of model residuals, with normality examined using Q–Q plots and homoscedasticity evaluated by residuals-versus-fitted plots. When the assumptions were met, Duncan’s multiple range test was used for post hoc comparisons. When the assumption of equal variances was violated, Welch’s ANOVA was applied, followed by the Games–Howell test. A value of *p* < 0.05 was considered statistically significant. All statistical analyses were conducted in SPSS 27, and figures were generated in Origin 2025.

## 3. Results

### 3.1. Glycation Reactivity: Color Change and Apparent Grafting

#### 3.1.1. Color Development

**Figure 1:**
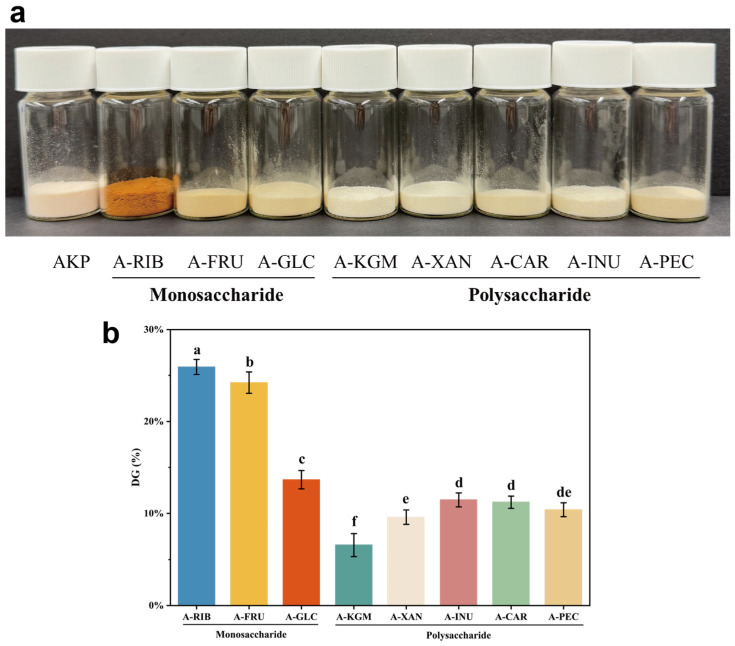
(**a**) Photographs of lyophilized samples of different glycosylating agents modified AKP. (**b**) DG values of different glycosylating agents modified AKP. Note: Data are expressed as mean ± SD (n = 5). Different lowercase letters indicate significant differences (*p* < 0.05). AKP, Antarctic krill protein; A-RIB, ribose-modified AKP; A-FRU, fructose-modified AKP; A-GLC, glucose-modified AKP; A-KGM, konjac glucomannan-modified AKP; A-XAN, xanthan gum-modified AKP; A-INU, inulin-modified AKP; A-CAR, carrageenan-modified AKP; A-PEC, pectin-modified AKP.

Color development is a rapid, integrative indicator of Maillard progression. Native AKP powder showed a slight pink hue, whereas glycosylation with monosaccharides yielded visibly darker powders than polysaccharide-modified samples (Figure 1a). Consistently, the total color difference (ΔE*) (Table 1)showed a clear separation between the two sugar classes: all monosaccharide conjugates exhibited markedly higher ΔE* values than the polysaccharide conjugates under identical heating conditions. Among the monosaccharides, A-RIB showed the most intense browning (ΔE* = 64.55), followed by A-FRU (31.90) and A-GLC (21.40). In contrast, polysaccharide conjugates remained close to the native AKP level (AKP, 12.87; A-KGM, 8.79; A-XAN, 9.26; A-CAR, 11.13; A-INU, 15.63; A-PEC, 16.90).

The pronounced browning of A-RIB is also consistent with the higher Maillard reactivity of pentoses, which accelerates the formation of reactive intermediates and brown pigments relative to hexoses. This pattern agrees with Li et al. (2025), who reported that monosaccharides with fewer carbon atoms promoted Maillard glycosylation more effectively, with color development following tetrose > pentose > hexose in sheep trotter gelatin systems [36]. Notably, the minimal color changes observed for most polysaccharide conjugates suggest slower and/or sterically constrained reactions, indicating that substantial functional improvements do not necessarily require pronounced browning. From an application perspective, this is advantageous because excessive browning can adversely affect product appearance and consumer acceptance.

#### 3.1.2. Degree of Glycosylation (DG)

During glycosylation, free amino groups on protein side chains react with carbonyl groups at the reducing ends of sugars; therefore, the extent of reaction can be tracked by the loss of OPA-reactive amino groups and expressed as an apparent DG [37]. All glycosylated samples exhibited higher DG than the control AKP (Figure 1b), confirming that both monosaccharides and polysaccharides successfully participated in Maillard-type modification. Consistent with the ΔE* results, monosaccharide-modified AKP showed significantly higher DG than polysaccharide-modified AKP (*p* < 0.05). This pattern is expected because small sugars can access reactive amino sites more readily [38], whereas long-chain polysaccharides may induce spatial site-blocking and partial shielding of reactive carbonyl groups, lowering effective collision frequency and slowing conjugation.

Within the monosaccharides, the DG trend mirrored browning (A-RIB > A-FRU ≳ A-GLC), supporting the view that Maillard progression in this system was strongly influenced by sugar size, structure and associated reactivity. Together, ΔE* and DG indicate that sugar type may help decouple reaction severity from downstream functionality. Due to their relatively small molecular size and relatively low steric hindrance, monosaccharides can achieve higher apparent degrees of grafting (DG), but they also induce more pronounced browning, consistent with the findings of Xu et al. [39]. However, the limited chain length of monosaccharides means that their contribution to steric stabilization and hydration of the modified protein is likely to be relatively weak.

In contrast, although polysaccharides showed lower DG values and milder color changes, their long-chain architecture may still contribute substantially to structural and functional tuning. In particular, bulky carbohydrate chains can act as spatial barriers that reduce close packing and re-aggregation of protein molecules [40,41]. Their abundant hydrophilic groups may also enhance water association around the conjugates, thereby promoting dispersion stability [42]. In addition, for some polysaccharides, especially those with bulky or charged chains, altered interfacial adsorption and rearrangement may contribute to improved interfacial behavior by favoring the formation of thicker and more stable interfacial layers [43]. Therefore, even with lower apparent grafting and weaker browning, polysaccharides can still induce meaningful structural and functional modifications, which is advantageous for food applications where color control is desirable.

### 3.2. Glycation Reactivity: Color Change and Apparent Grafting

#### 3.2.1. Particle Size Distribution

As shown in Table 2, native AKP exhibited the largest particle size, whereas glycosylation significantly reduced particle size across all treatments (*p* < 0.05). After modification, mean particle sizes decreased to 85–185 μm depending on the carbohydrate used. Among the treatments, A-PEC and A-XAN produced the smallest particles, while A-KGM and A-RIB remained comparatively larger. The reduction in particle size can be attributed to structural changes induced by Maillard-type glycation. Covalent attachment of carbohydrate moieties introduces additional hydrophilic groups onto protein side chains, which weakens hydrophobic aggregation and increases steric repulsion between protein molecules [44]. These effects facilitate better dispersion of protein particles in aqueous systems and reduce the formation of large aggregates.

Differences among sugars likely arise from variations in carbohydrate molecular size and structure [45,46,47]. Polysaccharides with extended chain conformations may provide stronger steric stabilization and hydration layers, which can effectively limit particle association. In contrast, monosaccharides generally promote faster glycation reactions (as reflected in the higher DG values), but their smaller molecular size provides less steric shielding against protein–protein interactions. Li et al. [48] reported that ribose-modified pea protein showed the smallest particle size and lowest polydispersity, likely due to its greater grafting extent. In the present AKP system, however, ribose did not produce the smallest particles. A plausible explanation is that the reaction conditions used here may have promoted stronger thermal denaturation and unfolding of AKP during modification, which can expose internal hydrophobic patches and partially offset the dispersing effect of glycation by favouring re-aggregation. This highlights that particle size reflects the net balance between glycation-induced hydration/steric stabilisation and heat-induced unfolding/association, and that the optimal carbohydrate choice may be system- and process-dependent.

#### 3.2.2. FTIR Analysis

**Figure 2:**
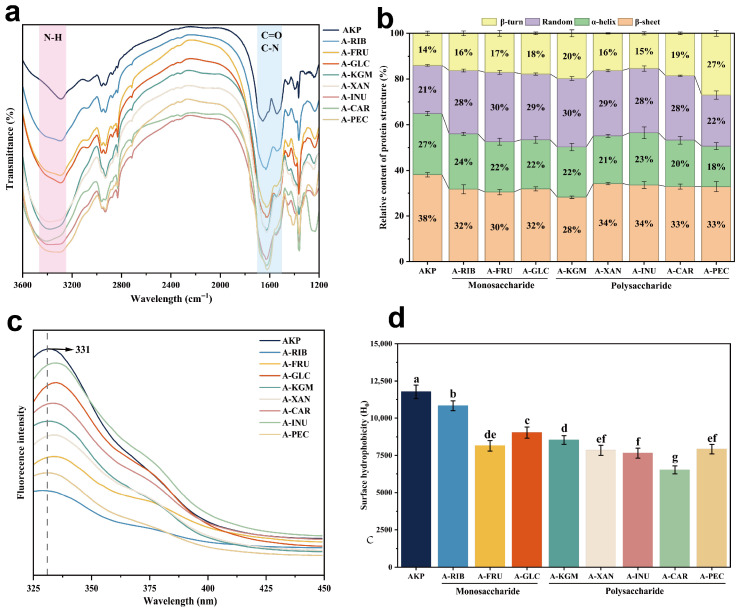
(**a**) FTIR spectra of AKP modified with different glycosylating agents. (**b**) Secondary structural changes in AKP modified by different glycosylation reagents. (**c**) Intrinsic fluorescence spectra of AKP modified by different glycosylating agents. (**d**) Surface hydrophobicity (H0) of AKP modified by different glycosylation agents. Note: Data are expressed as mean ± SD (n = 5). Different lowercase letters indicate significant differences (*p* < 0.05). AKP, Antarctic krill protein; A-RIB, ribose-modified AKP; A-FRU, fructose-modified AKP; A-GLC, glucose-modified AKP; A-KGM, konjac glucomannan-modified AKP; A-XAN, xanthan gum-modified AKP; A-INU, inulin-modified AKP; A-CAR, carrageenan-modified AKP; A-PEC, pectin-modified AKP.

FTIR spectroscopy was used to evaluate changes in protein secondary structure induced by glycosylation. In particular, the amide I band (1700–1600 cm^−1^), associated with C=O stretching, the amide II band (1600–1500 cm^−1^), related to C–N stretching and N–H bending, and the amide A band (~3000 cm^−1^), arising from N–H stretching, are sensitive to alterations in protein conformation [49]. As shown in Figure 2a, compared with native AKP, the glycosylated samples exhibited a noticeable decrease in intensity and broadening of the amide A band. Because the amide A region mainly reflects N–H stretching vibrations, this change indicates that glycosylation altered the hydrogen-bonding environment surrounding peptide N–H groups. Specifically, the covalent attachment of sugar moieties and the introduction of abundant hydroxyl groups likely replaced part of the original protein–protein hydrogen bonding with protein–sugar hydrogen bonding [50]. This hydrogen-bond rearrangement would generate a more heterogeneous microenvironment for N–H groups, thereby leading to band broadening, and has been widely regarded as evidence that glycation affects not only covalent grafting but also the intermolecular interaction network of proteins [26]. In addition, the weakening of absorption in the amide II region (1500–1550 cm^−1^) suggests partial disruption of ordered secondary structures and increased conformational flexibility. These structural changes are consistent with the reduced particle size and enhanced solubility observed after glycosylation. The introduction of carbohydrate chains likely disrupts intramolecular interactions and promotes a more open protein conformation, thereby improving dispersion behavior.

Amide I band deconvolution was used to quantify protein secondary structure changes (Figure 2b). Native AKP exhibited a highly ordered structure, with relatively high contents of β-sheet (38%) and α-helix (27%), while random coil (21%) and β-turn (14%) were comparatively low. After Maillard glycation, all conjugates showed a consistent trend of decreased ordered structures and increased disordered structures [51,52]. In monosaccharide-modified samples, α-helix and β-sheet decreased to 22–24% and 30–32%, while random coil increased to 28–30%, indicating disruption of intramolecular hydrogen bonds and partial unfolding toward flexible conformations [38]. This effect was more pronounced in polysaccharide-modified proteins due to their larger molecular weight and complex structures, which promoted chain unfolding and extension, while negatively charged polysaccharides further enhanced electrostatic repulsion and structural rearrangement [53]. Additionally, the reduction in α-helix content is associated with enhanced protein–protein interactions, which contribute to improved stability of proteins at oil–water interfaces, consistent with the functional results observed in subsequent protein studies [54,55].

#### 3.2.3. Fluorescence Spectroscopy

Intrinsic fluorescence spectroscopy was used to probe tertiary structural changes in AKP following glycosylation. As shown in Figure 2c, compared with native AKP, glycosylated samples exhibited a clear red shift in the maximum emission wavelength, indicating that aromatic residues (e.g., tryptophan and tyrosine) became more exposed to a polar environment as a result of protein unfolding [56]. This observation is consistent with the FTIR results, which suggested disruption of ordered structures and increased conformational flexibility. In addition, fluorescence intensity decreased after glycosylation, which may be attributed to the shielding effect of covalently attached sugar chains as well as fluorescence quenching during the Maillard reaction. Among the treatments, A-RIB showed the most pronounced quenching effect, consistent with its higher reactivity and degree of glycation [48,57].

#### 3.2.4. Surface Hydrophobicity

Surface hydrophobicity (H_0_), assessed using ANS fluorescence, decreased significantly in all glycosylated samples compared with native AKP (*p* < 0.05), indicating reduced apparent exposure of hydrophobic residues (Figure 2d). Although glycosylation and thermal treatment can induce partial unfolding—as supported by FTIR and fluorescence results—the exposed hydrophobic regions are likely masked by covalently attached carbohydrate chains [58]. Polysaccharide-modified proteins exhibited a more pronounced decrease in H_0_; these bulky sugar moieties introduce steric hindrance and hydration layers that restrict ANS binding, resulting in lower measured H_0_ values [59]. Monosaccharide-modified proteins exhibited higher glycation reactivity and degrees of grafting; however, under stronger reaction conditions (e.g., A-RIB), partial reaggregation or re-burial of hydrophobic regions may occur, reflecting a balance between unfolding-induced exposure and glycation-induced shielding.

#### 3.2.5. Microstructure by SEM

**Figure 3:**
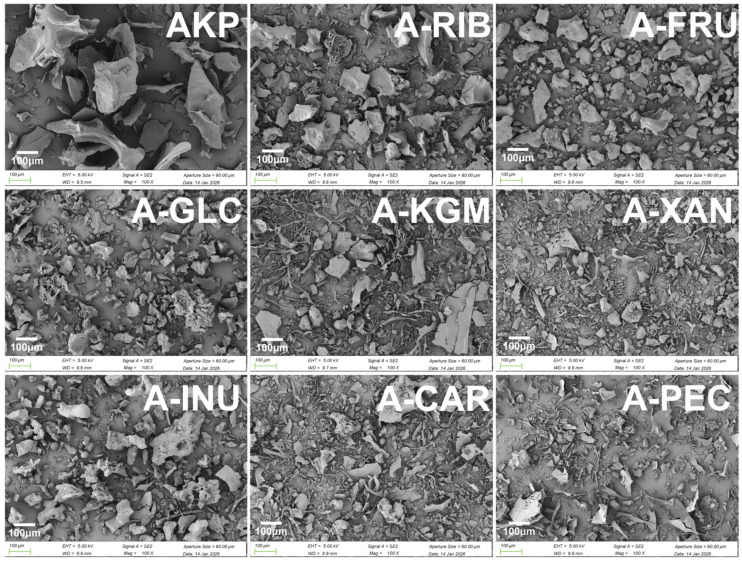
Glycosylated protein scanning electron microscope image. Note: The above SEM images were all observed and captured at 5 kV and 100× magnification. AKP, Antarctic krill protein; A-RIB, ribose-modified AKP; A-FRU, fructose-modified AKP; A-GLC, glucose-modified AKP; A-KGM, konjac glucomannan-modified AKP; A-XAN, xanthan gum-modified AKP; A-INU, inulin-modified AKP; A-CAR, carrageenan-modified AKP; A-PEC, pectin-modified AKP.

As shown in Figure 3, SEM images revealed that native AKP exhibited an irregular block-like morphology with a relatively smooth and compact surface, accompanied by large and heterogeneous particle sizes, consistent with previous reports [9]. Such a dense structure likely limits the accessibility of water molecules to internal hydrophilic groups, in agreement with the low solubility and large particle size observed for AKP. After glycosylation, the compact structure of AKP was disrupted, showing clear fragmentation and a reduction in particle size. However, it should be noted that SEM images mainly provide qualitative information, and direct comparison of particle size between samples is limited due to aggregation and resolution constraints. Therefore, particle size differences were primarily evaluated based on laser diffraction results (Table 2), which showed a clear reduction in D_[4,3]_ values after glycosylation.

Notably, the resulting microstructure depended strongly on the type of glycosylating agent [60]. Among the monosaccharide-modified samples, the quantitative data indicated that A-GLC had a relatively smaller particle size, whereas A-RIB and A-FRU remained comparatively larger. Among the polysaccharide-modified samples, A-CAR, A-XAN, and A-PEC tended to show smaller particle sizes and relatively looser aggregate morphologies, and in some areas partially fibrous or sheet-like features could also be observed. By contrast, A-INU remained comparatively larger, and its morphology was closer to that of some monosaccharide-modified systems. This may be related to the relatively low molecular weight of inulin compared with the other polysaccharides, which may limit the steric hindrance and hydration effects typically associated with larger polysaccharide chains. These observations are consistent with the trends in particle size, surface hydrophobicity, and solubility observed above.

### 3.3. Functional Properties

#### 3.3.1. Solubility

**Figure 4:**
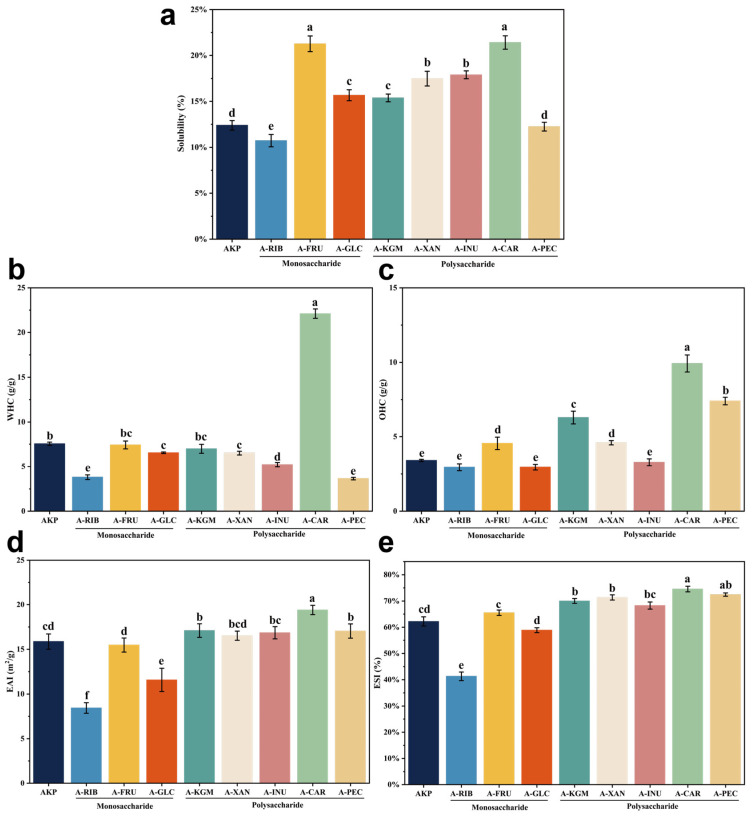
(**a**) Solubility of AKP modified with different glycosylating agents. (**b**) Water-holding capacity (WHC) of AKP modified by different glycosylating agents. (**c**) Oil-holding capacity (OHC) of AKP modified by different glycosylation agents. (**d**) Emulsifying activity index (EAI) of AKP modified by different glycosylation agents. (**e**) Emulsifying stability index (ESI) of AKP modified by different glycosylation agents. Note: Data are expressed as mean ± SD (n = 5). Different lowercase letters indicate significant differences (*p* < 0.05). AKP, Antarctic krill protein; A-RIB, ribose-modified AKP; A-FRU, fructose-modified AKP; A-GLC, glucose-modified AKP; A-KGM, konjac glucomannan-modified AKP; A-XAN, xanthan gum-modified AKP; A-INU, inulin-modified AKP; A-CAR, carrageenan-modified AKP; A-PEC, pectin-modified AKP.

Solubility is a key functional property of proteins and strongly influences their performance in food systems [26]. As shown in Figure 4a, native AKP exhibited low solubility (12.19%), which may be related to its compact structure and strong intermolecular interactions, as indicated by its large particle size and SEM observations.

After glycosylation, the solubility response depended strongly on carbohydrate structure. In most cases (A-FRU, A-GLC, A-KGM, A-XAN, A-INU, and A-CAR), the improvement in solubility is better understood as the result of a combined stabilization mechanism rather than a simple increase in hydrophilicity alone. At the molecular level, covalently attached carbohydrate chains may enhance water association around the protein and reduce direct protein–protein contacts, thereby suppressing aggregation [61,62]. At the supramolecular level, especially in polysaccharide-modified systems, extended carbohydrate chains can introduce steric constraints that hinder re-association of partially unfolded protein molecules and help maintain a more dispersed state [63]. In addition, changes in surface charge distribution induced by glycation may further enhance electrostatic repulsion, contributing to improved colloidal stability (e.g., A-XAN and A-CAR) [64].

By contrast, the reduced solubility observed in some samples suggests that destabilizing effects became dominant under the present reaction conditions. For A-RIB, the high reactivity of the pentose sugar likely promotes more extensive Maillard reaction and thermal aggregation, which may have offset the solubility gain expected from carbohydrate incorporation [48]. For A-PEC, electrostatically mediated protein–polysaccharide interactions may induce charge bridging under heating conditions, promoting the formation of compact aggregates [65]. Overall, the solubility of glycated AKP appears to be governed by the balance between glycation-induced stabilization, including hydration and steric effects, and competing aggregation pathways generated during thermal treatment, rather than by hydrophilicity alone.

#### 3.3.2. Water- and Oil-Holding Capacity

Water-holding capacity (WHC) and oil-holding capacity (OHC) reflect the ability of proteins to interact with aqueous and lipid phases, respectively, and are important indicators of functionality in food systems [66]. As shown in Figure 4b,c, glycosylation markedly altered both WHC and OHC, with carrageenan-modified AKP (A-CAR) exhibiting the highest values for both capacities.

For WHC, increases were observed in several samples, whereas A-RIB, A-INU, and A-PEC showed reduced water retention compared with native AKP. The enhanced WHC can be attributed to the introduction of hydrophilic carbohydrate chains and the more open, dispersed structures observed in Section 3.2, which provide additional sites for water binding. In contrast, reduced WHC in some samples may result from excessive glycation or heat-induced aggregation, which limits water accessibility despite partial unfolding. For OHC, most glycosylated samples exhibited significant increases (*p* < 0.05), except A-RIB, A-GLC, and A-INU. The improvement in OHC is likely associated with structural rearrangements that expose hydrophobic regions and facilitate interactions with lipid molecules. At the same time, the presence of carbohydrate chains may enhance oil retention by forming a more stable protein–lipid matrix [67,68].

Overall, these results suggest that WHC and OHC are governed by a balance between hydrophilic modification (favoring water binding) and structural rearrangement/aggregation (influencing oil binding). Notably, polysaccharide-modified samples, particularly A-CAR, showed superior performance, which is consistent with their looser microstructure, lower surface hydrophobicity, and improved dispersion characteristics.

#### 3.3.3. Emulsifying Properties (EAI and ESI)

Emulsifying properties, expressed as the emulsifying activity index (EAI) and emulsion stability index (ESI), are shown in Figure 4d,e. Glycosylation with different carbohydrates led to distinct changes in emulsifying performance. Overall, monosaccharide-modified proteins exhibited little improvement or even a reduction in EAI, with A-RIB and A-GLC showing a significant decrease (*p* < 0.05). Similarly, ESI values of monosaccharide-modified samples showed no significant enhancement compared with native AKP. These results suggest that, despite their higher glycation reactivity, monosaccharides did not improve interfacial functionality and may even impair protein adsorption at the oil–water interface [69].

In contrast, polysaccharide-modified proteins showed clear improvements in both EAI and ESI, with A-CAR exhibiting the most pronounced enhancement (AKP vs. A-CAR: EAI 15.87 → 19.40 m^2^/g; ESI 62.21% → 74.57%). This improvement can be attributed to the combined effects of enhanced solubility, reduced particle size, and more dispersed microstructures observed in Section 3.2, which facilitate rapid adsorption and uniform distribution at the interface. In addition, the presence of long-chain polysaccharides increases interfacial layer thickness and provides steric stabilization, thereby improving resistance to droplet aggregation and coalescence.

The contrasting behavior between monosaccharide- and polysaccharide-modified proteins highlights that glycation reactivity alone does not determine emulsifying performance. Instead, interfacial functionality is governed by a balance between molecular flexibility, surface hydrophobicity, and steric stabilization. Polysaccharide conjugation, particularly with carrageenan, appears to optimize this balance by enhancing both interfacial adsorption and emulsion stability [70].

## 4. Conclusions

This study demonstrates that polysaccharide-mediated glycosylation is more effective than monosaccharide modification in improving the functional properties of Antarctic krill protein (AKP). Despite lower grafting degrees, polysaccharides promoted favorable structural changes, including reduced particle size and lower surface hydrophobicity, which enhanced solubility, water- and oil-holding capacity, and emulsifying properties. Among them, carrageenan showed the most pronounced improvement. Overall, the functional performance of glycosylated proteins depends on the balance between molecular-scale reactivity and mesoscale structural effects (such as spatial stability and hydration). This study investigated the structural and functional properties of glycosylated Antarctic krill protein (AKP) in simple aqueous model systems. The performance of modified AKP in real food matrices and during processing remains to be validated. Future work should focus on evaluating its behavior in food systems and under industrial processing conditions to support practical applications.

## Figures and Tables

**Table 1:** Color changes in AKP modified by different glycosylation reagents.

Sample	ΔE*
AKP	12.87 ± 0.43 ^f^
A-RIB	64.55 ± 0.38 ^a^
A-FRU	31.90 ± 0.39 ^b^
A-GLC	21.40 ± 0.28 ^c^
A-KGM	8.79 ± 0.35 ^i^
A-XAN	9.26 ± 0.30 ^h^
A-INU	15.63 ± 0.34 ^e^
A-CAR	11.13 ± 0.38 ^g^
A-PEC	16.90 ± 0.38 ^d^

Note: Data are expressed as mean ± SD (n = 5). Different lowercase letters indicate significant differences (*p* < 0.05). AKP, Antarctic krill protein; A-RIB, ribose-modified AKP; A-FRU, fructose-modified AKP; A-GLC, glucose-modified AKP; A-KGM, konjac glucomannan-modified AKP; A-XAN, xanthan gum-modified AKP; A-INU, inulin-modified AKP; A-CAR, carrageenan-modified AKP; A-PEC, pectin-modified AKP.

**Table 2:** Particle size of AKP modified by different glycosylation reagents.

Sample	D_[4,3]_ (μm)
AKP	316.78 ± 5.43 ^a^
A-RIB	186.11 ± 2.67 ^b^
A-FRU	150.00 ± 2.40 ^c^
A-GLC	108.33 ± 1.33 ^e^
A-KGM	184.22 ± 2.36 ^b^
A-XAN	90.24 ± 3.10 ^g^
A-INU	118.67 ± 2.33 ^d^
A-CAR	104.22 ± 0.69 ^f^
A-PEC	85.28 ± 0.73 ^g^

Note: D_[4,3]_ represents the volume-weighted mean diameter. Data are expressed as mean ± SD (n = 5). Different lowercase letters indicate significant differences (*p* < 0.05). AKP, Antarctic krill protein; A-RIB, ribose-modified AKP; A-FRU, fructose-modified AKP; A-GLC, glucose-modified AKP; A-KGM, konjac glucomannan-modified AKP; A-XAN, xanthan gum-modified AKP; A-INU, inulin-modified AKP; A-CAR, carrageenan-modified AKP; A-PEC, pectin-modified AKP.

## Data Availability

The raw data supporting the conclusions of this article will be made available by the authors on request.
